# Footstep Energy Harvesting with the Magnetostrictive Fiber Integrated Shoes

**DOI:** 10.3390/ma12132055

**Published:** 2019-06-26

**Authors:** Hiroki Kurita, Kenichi Katabira, Yu Yoshida, Fumio Narita

**Affiliations:** Department of Materials Processing, Graduate School of Engineering, Tohoku University, Aoba-yama 6-6-02, Sendai 980-8579, Japan

**Keywords:** magnetostrictive, energy harvesting, wearable

## Abstract

Wearable energy harvesting devices attract attention as the devices provide electrical power without inhibiting user mobility and independence. While the piezoelectric materials integrated shoes have been considered as wearable energy harvesting devices for a long time, they can lose their energy harvesting performance after being used several times due to their brittleness. In this study, we focused on Fe–Co magnetostrictive materials and fabricated Fe–Co magnetostrictive fiber integrated shoes. We revealed that Fe–Co magnetostrictive fiber integrated shoes are capable of generating 1.2 µJ from 1000 steps of usual walking by the Villari (inverse magnetostrictive) effect. It seems that the output energy is dependent on user habit on ambulation, not on their weight. From both a mechanical and functional point of view, Fe–Co magnetostrictive fiber integrated shoes demonstrated stable energy harvesting performance after being used many times. It is likely that Fe–Co magnetostrictive fiber integrated shoes are available as sustainable and wearable energy harvesting devices.

## 1. Introduction

Miniaturization technology of electrical devices has allowed the development of various portable devices, such as watches, smartphones, etc. However, further downsizing of electrical devices, which is required with advances in electronic technology, has not yet been achieved because of the difficulty to downsize batteries for portable devices. Therefore, energy-harvesting devices, which generate electrical power from mechanical phenomena, attract attention for developing battery-free systems. Wearable energy harvesting devices have especially attracted a lot of interest as they can provide electrical power and ensure user mobility and independence [1].

Piezoelectric materials have been considered as wearable energy harvesting devices [2,3], and mainly integrated into shoes to harvest a large amount of mechanical energy [4,5,6,7,8,9]. For example, Turkman et al. have reported 1.4 mW of generated power by the applied mass of 90 kg [9]. However, it is well known that piezoelectric materials generally have low impact resistance and are easy to depolarize. Hence, piezoelectric materials integrated shoes have a high probability of fracturing, having electric fatigue, and becoming dysfunctional immediately; however, they show good energy harvesting performance for a temporary period of time [10].

Magnetostrictive materials have been considered as other potential candidate materials for energy harvesting [11,12]. Vibration energy harvesting by magnetostrictive materials allows one to obtain a representative high output electric current compared to piezoelectric materials [13]. In recent years, a new structure of magnetostrictive TbDyFe alloy (Terfenol-D) generator for harvesting the rotation of the human knee joint was presented [14]. However, the typical magnetostrictive materials, Terfenol-D and Galfenol alloys, are extremely brittle despite having outstanding magnetostriction (respectively 800–1200 ppm and 120–240 ppm [12]). Therefore, we have recently focused on iron cobalt (Fe–Co) magnetostrictive materials, which have acceptable toughness and are inexpensive [15,16,17]. It seems that Fe–Co magnetostrictive materials endure cyclic loads, and are a prime candidate material for an energy harvesting among magnetostrictive materials. Furthermore, the energy harvesting performance of magnetostrictive fiber integrated shoes has not yet been reported to the best of our knowledge. In this study, we fabricated Fe–Co magnetostrictive fiber integrated shoes and evaluated the output energy of footstep energy harvesting during ambulation activities.

## 2. Experimental Procedure

**Figure 1:**
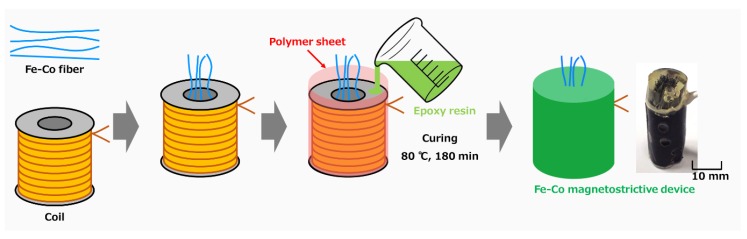
Schematic illustration of the preparation process of Fe–Co magnetostrictive device.

**Figure 2:**
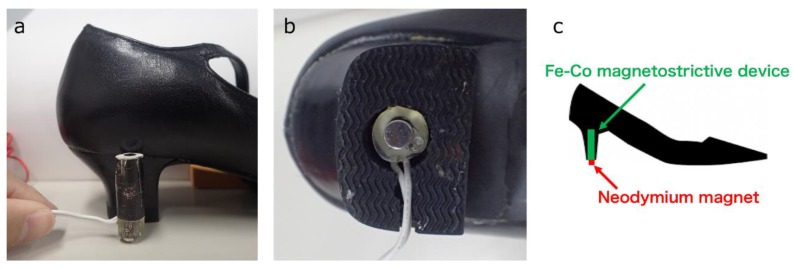
Integrated position of Fe–Co magnetostrictive device into a hollowed heel of the left pump. (**a**) side view, (**b**) bottom view, and (**c**) schematic illustration of Fe–Co magnetostrictive fiber integrated shoe.

Figure 1 shows the preparation process of the Fe–Co magnetostrictive device. The Fe–Co continuous fibers were prepared by drawing, and the composition is Fe_29_Co_71_. 125 Fe–Co fibers with a diameter of 0.2 mm and a solenoid coil with 1050 turns (CA05310180, Takaha Kiko Co., Ltd., Iizuka, Fukuoka, Japan) were prepared as starting materials. The coil was covered with a polymer sheet and Fe–Co fibers were inserted into the center hole of the coil. After that, a mixed solution of Bisphenol F and curing agent were casted into the polymer sheet and cured at 80 °C for 180 min to fabricate the Fe–Co magnetostrictive device. The mixing weight ratio of Bisphenol F: Curing agent was determined to be 100:55. The Fe–Co magnetostrictive device was polished to obtain a diameter of 12 mm and a height of 34 mm, and integrated into a hollowed heel of the left pump, as shown in Figure 2. The volume fractions of Fe–Co fiber and other parts (epoxy matrix and solenoid coil) in the device were approximately 33% and 67%, respectively. The magnetostrictive coefficient of the Fe–Co magnetostrictive device ($d_{33}^{c}$) is expected to become smaller than that of the original Fe–Co fiber ($d_{33}^{f}$) because the coefficient of the device is expressed simply by $d_{33}^{c}$ = *v*^f^ $d_{33}^{f}$, where *v*^f^ (=0.33) is the volume fraction of the Fe–Co fiber. A neodymium magnet with the surface magnetic induction of 360 mT was fixed at the bottom side of the Fe–Co magnetostrictive device, to orient the magnetization direction of Fe–Co fibers.

**Figure 3:**
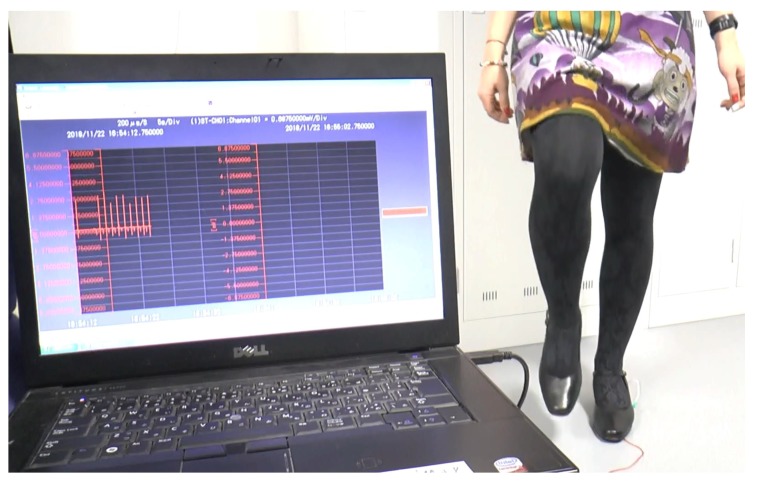
Output power evaluation appearance of Fe–Co magnetostrictive fiber integrated shoes during the usual walking of subject 1.

The Fe–Co magnetostrictive device was connected to a resistance of 20 Ω, which agrees with the value of the device (19.7 Ω) to optimize output voltage and a data logger in parallel. Note that the input resistance value of the data logger was 14.5 MΩ. The output power was evaluated from a voltage and a resistance during two different ambulation activities of two subjects. Figure 3 shows the output power evaluation appearance during the usual walking of subject 1.

## 3. Theory

Let us now consider the system using rectangular Cartesian coordinates *x_i_* (O-*x*_1_, *x*_2_, *x*_3_). The easy axis for the magnetization of a magnetostrictive fiber is along the length direction (*x*_3_-direction) [18]. For the magnetostrictive fiber, the changes of magnetic induction are decided by the stress amplitude applied in the direction of the fiber. The constitutive equations of the one-dimensional magnetostrictive fiber can be written as [16]

(1) $$\varepsilon_{33}=s_{33}\sigma_{33}+d_{33}^{f}H_{3}=s_{33}\sigma_{33}+d_{33}^{m}\left(\frac{B_{0}}{\mu_{33}}\right)+m_{33}{\left(\frac{B_{0}}{\mu_{33}}\right)}^{2}$$

(2) $$B_{3}=d_{33}^{m}\sigma_{33}+m_{33}\left(\frac{B_{0}}{\mu_{33}}\right)\sigma_{33}+B_{0}$$

where $\sigma_{33}$ and $\varepsilon_{33}$ are the stress and strain components, $B_{3}$ and $H_{3}$ are the magnetic induction and magnetic field intensity components, $s_{33}$, $d_{33}^{m}$, $m_{33}$, and $\mu_{33}$ are the elastic compliance, piezomagnetic constant, second-order magnetoelastic constant and magnetic permeability, respectively, and $B_{0}$ is the magnetic bias field. The output power is given using the resistance R as

(3) $$P_{\mathrm{out}}=\frac{V_{\mathrm{out}}^{2}}{R}$$

where the output voltage $V_{\mathrm{out}}$ for the magnetostrictive fiber is obtained as

(4) $$V_{\mathrm{out}}=-NA\frac{dB_{3}}{dt}$$

In Equation (4), *N* is the number of turns in the search coil, A is the cross-sectional area of all magnetostrictive fibers and *t* is the time. Substituting Equation (2) into Equation (4) gives

(5) $$V_{\mathrm{out}}=-NA\left[d_{33}+\left(\frac{m_{33}}{\mu_{33}}\right)B_{0}\right]\frac{d\sigma_{33}}{dt}$$

It is clear that the output voltage is proportional to the time derivative of the stress in the fiber. This mechanism differs from the piezoelectric material. That is, the output voltage of the piezoelectric material is proportional to the electric field amplitude associated with the stress amplitude [3].

## 4. Results and Discussion

**Figure 4:**
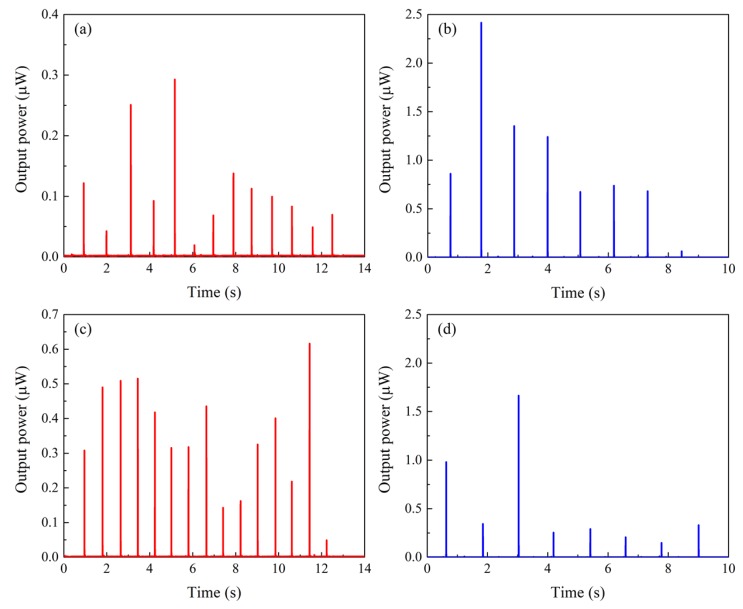
Output power obtained by Fe–Co magnetostrictive fiber integrated shoes; (**a**) during the usual walking of subject 1, (**b**) during the usual walking of subject 2, (**c**) during the walking with legs raised higher in subject 1, and (**d**) during the walking with legs raised higher in subject 2.

**Figure 5:**
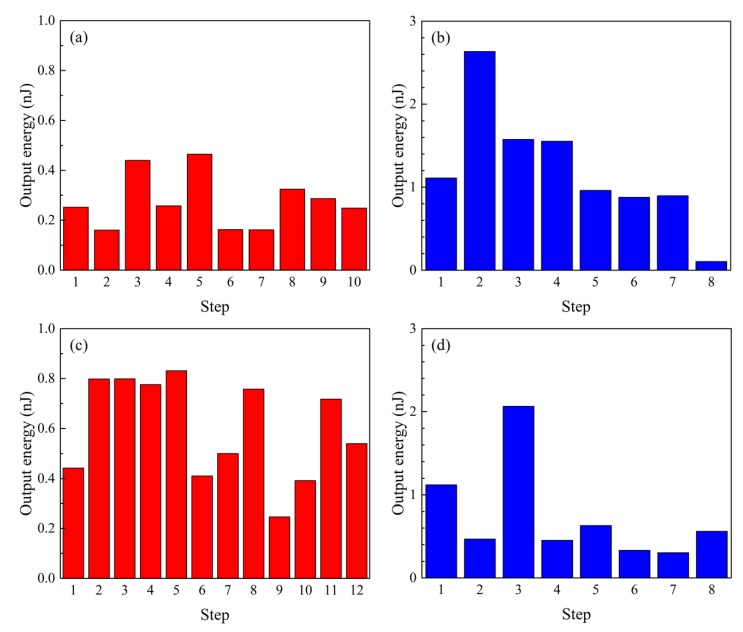
Output energy obtained by Fe–Co magnetostrictive fiber integrated shoes; (**a**) during the usual walking of subject 1, (**b**) during the usual walking of subject 2, (**c**) during walking with legs raised higher in subject 1, and (**d**) during the walking with legs raised higher in subject 2.

Figure 4 shows the output power obtained by Fe–Co magnetostrictive fiber integrated shoes. The output power was calculated by Equation (3). The subjects walked at a velocity of 1 step/s (1 Hz). The output power was obtained when the left foot of subjects contacted to the floor. This result clearly shows that this power generation was attributed to the Villari (inverse magnetostrictive) effect. Figure 5 shows the output energy. Although there was no correlation between output energy and each step, the output energy during the usual walking of subject 2 (Figure 5b) was qualitatively larger than that of subject 1 (Figure 5a). Note that subject 1 and 2 have almost the same weight, therefore it is indicated that the output energy is not dependent on the weight. The output energy during the usual walking of subject 1 (Figure 5a) increased when she raised her leg higher (Figure 5c). It seems that larger impacts at the heel of the pump generate a larger output energy. In fact, although the magnetic induction is proportional to the stress (referred to as the Villari effect in Equation (2)), the voltage depends on the time derivative of the magnetic induction, i.e., stress-rate (see Equation (5)). Hence, the output power (proportional to the square of the voltage) is related to the speed of the heel rather than the weight. On the other hand, the output energy during the usual walking of subject 2 decreased when she raised her leg higher (Figure 5d). Moreover, a larger output energy was obtained when she walked at a velocity of 2 steps/s (2 Hz) without raising her legs higher (i.e., when the foot of subject 1 was contacted faster than her usual walk). Consequently, it is implied that the output energy is dependent on the subject’s type of ambulation, not on the weight of subjects.

The maximum output energy achieved 2.6 nJ, and the instantaneous peak power was 2.4 µW. It is also capable of generating 1.2 µJ from 1000 steps of usual walking. In any case, it seems that Fe–Co magnetostrictive fiber integrated shoes generate low maximum output power, and it is necessary to generate larger output power for the practical realization of Fe–Co magnetostrictive fiber integrated shoes. The size of the coils dominate the output power from magnetostrictive materials, however, magnetostrictive devices should be small enough to be embedded inside shoes. It is likely that an important challenge in the coming years will be device size reduction as nanotechnology becomes increasingly prevalent. In addition, the magnetostrictive device was designed to fit into the heel of a woman’s shoe. When the device is embedded in another shoe (e.g., a flat shoe), we have to consider a different design of the magnetostrictive device. For example, one conceivable design for a flat shoe is to adapt the configuration to the sole of a flat shoe, utilizing its flexural deformation.

As mentioned above, general piezoelectric ceramics and typical magnetostrictive alloys are brittle. Therefore, these materials should lose their energy harvesting performance after being used several times or by an unheralded impact, even if they demonstrate outstanding energy harvesting for a temporary period of time. However, the Fe–Co magnetostrictive device did not break after 1 million compression tests (not shown here), whereas the coil was slightly damaged. While a fatigue test is required to investigate the accurate durability of Fe–Co magnetostrictive fiber integrated shoes, it is likely that Fe–Co magnetostrictive fiber integrated shoes permanently exercise an energy harvesting performance. From not only the mechanical point of view but also the functional one, Fe–Co magnetostrictive fiber integrated shoes have an advantage compared with piezoelectric materials integrated shoes. For piezoelectric materials, a polarization treatment is required to obtain excellent piezoelectric properties [3]. On the other hand, magnetostrictive materials do not require a polarization treatment; they provide stable magnetostrictive properties (i.e., energy harvesting performance). The Fe–Co magnetostrictive fiber integrated shoes used in this study actually demonstrated stable magnetostrictive properties. Hence, it seems that Fe–Co magnetostrictive fiber integrated shoes can be sustainable and wearable energy harvesting devices.

To achieve more stable and functional energy harvesting performance, we should explore every possibility for the best design of the shoes hereafter. For instance, it is necessary to consider the effect of a bias magnet interfering with the surface where the wearer walks, or the impact damage to a permanent magnet or electromagnet coil for biasing the magnetostrictive material.

## 5. Conclusions

The footstep energy harvesting output power and energy of Fe–Co magnetostrictive fiber integrated shoes were evaluated during two different ambulation activities of two subjects. It was revealed that output power and energy were obtained during the ambulation activities generation by the Villari effect.

Despite subject 1 and 2 having almost the same weight, the output energies from their ambulation activities were extremely different. Furthermore, the output energy increased when the subjects applied larger impacts at the heel of the pump. Therefore, it was implied that the output energy is dependent on the habit of subjects on ambulation, not on their weight. Especially the impact when the pump contacts on the floor seems a dominant factor.

From both the mechanical and functional point of view, Fe–Co magnetostrictive fiber integrated shoes demonstrated stable magnetostrictive properties (i.e., energy harvesting performance). We believe that Fe–Co magnetostrictive fiber integrated shoes can be sustainable and wearable energy harvesting devices, with the improvement of maximum output power and energy by optimizing the magnetostrictive devices.

## References

[1] Ylli K., Hoffmann D., Willmann A., Becker P., Folkmer B., Manoli Y. (2015). Energy harvesting from human motion: Exploiting swing and shock excitations. Smart Mater. Struct..

[2] Narita F., Nagaoka H., Wang Z. (2019). Fabrication and impact output voltage characteristics of carbon fiber reinforced polymer composites with lead-free piezoelectric nano-particles. Mater. Lett..

[3] Wang Z., Narita F. (2019). Corona poling conditions for barium titanate/epoxy composites and their unsteady wind energy harvesting potential. Adv. Eng. Mater..

[4] Mateu L., Moll F. (2006). Appropriate charge control of the storage capacitor in a piezoelectric energy harvesting device for discontinuous load operation. Sens. Actuators A.

[5] Rocha J.G., Goncalves L.M., Rocha P.F., Silva M.P., Lanceros-Mendez S. (2010). Energy harvesting from piezoelectric materials fully integrated in footwear. Trans. Ind. Electr..

[6] Alumusallam A., Torah R.N., Zhu D., Tudor M.J., Beeby S.P. (2013). Screen-printed piezoelectric shoe-insole energy harvester using an improved flexible PZT-polymer composites. J. Phys. Conf. Ser..

[7] Jung W.S., Lee M.J., Kang M.G., Moon H.G., Yoon S.J., Baek S.H., Kang C.Y. (2015). Powerful curved piezoelectric generator for wearable applications. Nano Energy.

[8] Kalantarian H., Sarrafzadeh M. (2016). Pedometers without batteries: An energy harvesting shoe. Sens. J..

[9] Turkman A.C., Celik C. (2018). Energy harvesting with the piezoelectric material integrated shoe. Energy.

[10] Siddiqui S., Kim D.-I., Roh E., Duy L.T., Trung T.Q., Nguyen M.T., Lee N.-E. (2016). A durable and stable piezoelectric nanogenerator with nanocomposite nanofibers embedded in an elastomer under high loading for a self-powered sensor system. Nano Energy.

[11] Deng Z., Dapino M.J. (2017). Review of magnetostrictive vibration energy harvesters. Smart Mater. Struct..

[12] Narita F., Fox M. (2018). A review on piezoelectric, magnetostrictive, and magnetoelectric materials and device technologies for energy harvesting applications. Adv. Eng. Mater..

[13] Yang Z.J., Nakajima K., Onodera R., Tayama T., Chiba D., Narita F. (2018). Magnetostrictive clad steel plates for high-performance vibration energy harvesting. Appl. Phys. Lett..

[14] Yan B., Zhang C., Li L. (2018). Magnetostrictive energy generator for harvesting the rotation of human knee joint. AIP Adv..

[15] Narita F. (2017). Inverse magnetostrictive effect in Fe_29_Co_71_ wire/polymer composites. Adv. Eng. Mater..

[16] Narita F., Katabira K. (2017). Stress-rate dependent output voltage for Fe_29_Co_71_ magnetostrictive fiber/polymer composites: Fabrication, experimental observation and theoretical prediction. Mater. Trans..

[17] Katabira K., Yoshida Y., Masuda A., Watanabe A., Narita F. (2018). Fabrication of Fe-Co magnetostrictive fiber reinforced plastic composites and their sensor performance evaluation. Materials.

[18] Yang Z.J., Nakajima K., Jiang L., Kurita H., Murasawa G., Narita F. (2019). Design, fabrication and evaluation of metal-matrix lightweight magnetostrictive fiber composites. Mater. Des..

